# Octopamine modulates activity of neural networks in the honey bee antennal lobe

**DOI:** 10.1007/s00359-013-0805-y

**Published:** 2013-05-17

**Authors:** Julia Rein, Julie A. Mustard, Martin Strauch, Brian H. Smith, C. Giovanni Galizia

**Affiliations:** 1 Neurobiologie, Universität Konstanz, 78457 Constance, Germany; 2 School of Life Sciences, Arizona State University, P.O. Box 874501, Tempe, AZ 85287 USA

**Keywords:** Olfaction, Insects, Octopamine, Plasticity, Calcium imaging

## Abstract

Neuronal plasticity allows an animal to respond to environmental changes by modulating its response to stimuli. In the honey bee (*Apis mellifera*), the biogenic amine octopamine plays a crucial role in appetitive odor learning, but little is known about how octopamine affects the brain. We investigated its effect in the antennal lobe, the first olfactory center in the brain, using calcium imaging to record background activity and odor responses before and after octopamine application. We show that octopamine increases background activity in olfactory output neurons, while reducing average calcium levels. Odor responses were modulated both upwards and downwards, with more odor response increases in glomeruli with negative or weak odor responses. Importantly, the octopamine effect was variable across glomeruli, odorants, odorant concentrations and animals, suggesting that the octopaminergic network is shaped by plasticity depending on an individual animal’s history and possibly other factors. Using RNA interference, we show that the octopamine receptor AmOA1 (homolog of the *Drosophila* OAMB receptor) is involved in the octopamine effect. We propose a network model in which octopamine receptors are plastic in their density and located on a subpopulation of inhibitory neurons in a disinhibitory pathway. This would improve odor-coding of behaviorally relevant, previously experienced odors.

**Electronic supplementary material:**

The online version of this article (doi:10.1007/s00359-013-0805-y) contains supplementary material, which is available to authorized users.

## Introduction

Forager honey bees must learn to associate floral odors with nectar and pollen rewards that their colony needs for survival. This learning ability has been extensively studied using laboratory procedures that allow for investigation of physiological bases that underlie olfactory learning and memory (Menzel and Giurfa 2001). These studies suggest that the biogenic amine octopamine (OA) represents the occurrence of sucrose in brain networks that process olfactory inputs. Octopaminergic cells extend throughout the brain, including regions important for olfactory learning such as the mushroom bodies (MB), antennal lobes (AL) and subesophageal ganglion (Kreissl et al. 1994; Monastirioti 1999; Sinakevitch et al. 2005). One set of octopaminergic neurons of particular interest is the ventral unpaired medial (VUM) cells. These cells have cell bodies along the ventral midline in the brain’s subesophageal ganglion and receive afferent input from sucrose sensitive taste receptor neurons on the honey bee’s mouthparts (Schröter et al. 2007). Electrophysiological recordings from one particular VUM cell (VUMmx1) have shown that its response to odor changes after that odor has been associated with sucrose reinforcement in a way that produces robust associative conditioning (Hammer 1993, 1997). Moreover, injection of OA into the brain is sufficient to replace sucrose reinforcement (Hammer and Menzel 1998), and disruption of OA pathways via RNA interference or pharmacological treatment blocks associative conditioning (Farooqui et al. 2003). Therefore, OA release is important for the formation of associative memory for an odor associated with sucrose reinforcement. Because of the broad distribution of VUM neuron arborizations (Schröter et al. 2007), it is likely that OA release drives plasticity in several different areas of the brain, such as in the AL (Hammer 1997).

Neural networks in the AL provide the first synaptic contact between afferent sensory inputs from olfactory sensory cells with interneurons in the brain. Axons from sensory cells that express the same receptor, and hence respond to the same odorants, converge in the AL to the same spatial position to form a glomerulus (Galizia and Rössler 2010). Each glomerulus is innervated by dendrites of 3–5 projection neurons (PN), which send axon outputs to represent olfactory information in other areas of the brain (Mobbs 1985). Calcium-imaging studies of odor-driven PN responses have revealed complex spatial and temporal properties that represent the identity and concentration of odorants (Sachse and Galizia 2003; Locatelli et al. 2013). The spatiotemporal patterns that represent an odor in the AL also change when that odor is associated with sucrose reinforcement (Fernandez et al. 2009). Specifically, the patterns for an odor associated with sucrose become significantly more distinct from a different odor explicitly not associated with sucrose (Rath et al. 2011). Therefore, one potential function of plasticity in the AL may be to make odors that are involved in finding floral rewards more detectable and discriminable from less relevant background odors (Smith et al. 2006; Riffell et al. 2013). As a result, each individual honey bee would have a different connectivity network in the AL that reflects its unique set of olfactory experiences. OA release by VUMmx1 may underlie this plasticity because fine OA positive processes from VUM innervate most if not all of the AL glomeruli.

OA acts via binding to different G protein coupled receptors that regulate intracellular levels of cyclic AMP or calcium (Evans and Maqueira 2005). A number of distinct OA receptor subtypes have been cloned and characterized from *Drosophila* (Han et al. 1998; Balfanz et al. 2005; Maqueira et al. 2005). *Drosophila* OA receptors cluster into two classes (Evans and Maqueira 2005): One class is the Octα/OAMB receptor class, which consists of one gene with two splice variants: the OAMB-AS/DmOA1A/DmOctα1A receptor and the OAMB-K3/DmOA1B/DmOctα1B receptor. Receptors in this group act to regulate intracellular calcium levels via IP_3_ activation by PLC (Balfanz et al. 2005; Hoff et al. 2011). The other *Drosophila* OA receptors make up the Octβ receptor class, which when stimulated act to increase intracellular cAMP levels (Maqueira et al. 2005). To date, only one OA receptor (AmOA1) from the Octα receptor class has been cloned and characterized from honey bee (Grohmann et al. 2003), although the honey bee genome also contains several more OA receptor homologs that are members of the Octβ receptor class (Evans and Maqueira 2005). When expressed in HEK cells, activation of AmOA1 receptors by OA leads to oscillations of intracellular Ca^2+^ levels and a relatively small increase in cAMP levels (Grohmann et al. 2003). AmOA1 receptors are present throughout the brain, including in the mushroom bodies and ALs (Sinakevitch et al. 2011). Downregulation of AmOA1 via RNA interference significantly reduces olfactory learning (Farooqui et al. 2003), suggesting that AmOA1 receptors are an important part of the OA reinforcement pathway. OA could potentially have direct effects on several different cell types in the AL. In addition to sensory axon terminals and PNs, there are a number of interneurons that differ in morphology with regard to immunoreactivity for different neurotransmitters or peptides (Kreissl et al. 2010). A subset of GABAergic interneurons express AmOA1 receptors and are therefore targets for OA modulation (Sinakevitch et al. 2011).

Here we use calcium imaging of PN activity to perform a network level analysis of the effect of OA on odor representations in the AL with the ultimate objective of understanding how OA may target components of the network. Much of the specific synaptic connectivity between the different cell types within and among glomeruli is unknown. Yet we can predict that the effects of OA treatment on this network will be complex given the number of different cell types and the presence of AmOA1 receptors on the GABAergic interneurons. For example, we find that the calcium response from PNs in a glomerulus may be potentiated by OA in the presence of one odor. The same PNs may be inhibited in the presence of a different odor, which may only weakly excite these PNs, particularly if inhibition from a different glomerulus is potentiated by OA. Furthermore, we find a high variability across individuals, suggesting that OA is indeed involved in the individual learning history of the bee. Data such as these will be necessary for understanding in more detail the circuitry in the AL as well as how OA-mediated plasticity alters the network to adapt to changing contingences among odors. It is interesting to compare these findings to the situation in the mammalian olfactory bulb, in which similar coding and plasticity mechanisms have been described (Hildebrand and Shepherd 1997; Wilson 2008; Leinwand and Chalasani 2011), but where multiple modulators, including acetyl choline, norepinephrine and serotonin may exert related plasticity effects on olfactory networks (Fletcher and Chen 2010).

## Materials and methods

### Animals

Forager honey bees (*Apis mellifera carnica*) were collected in the morning at the hive entrance. Pollen and nectar foragers vary in a number of different physiological measures including sucrose sensitivity and learning (Page et al. 1998; Scheiner et al. 2005; Wright et al. 2007; Drezner-Levy et al. 2009), therefore, to reduce variability across animals, only pollen foragers were collected.

Bees were immobilized by cooling and then individually restrained in harnesses. The head was fixed to the harness with soft dental wax (Kerr, Sybron Dental Specialities) such that the bees could move their antennae and proboscis freely. During the experimental procedure, bees were kept at room temperature in plastic boxes with moist tissue and fed to satiation at least two times a day with 1 M sucrose solution.

### Projection neuron staining and calcium imaging

**Fig. 1:**
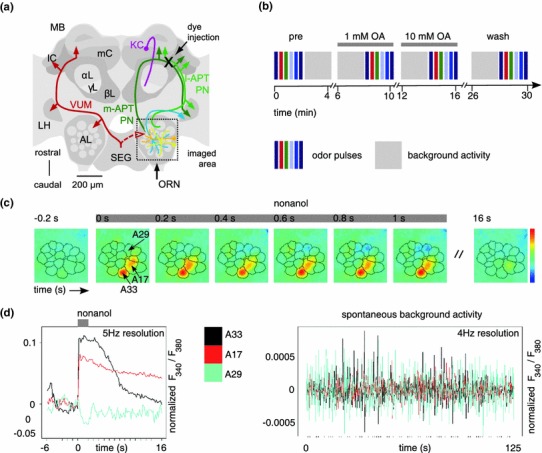
The experimental procedure. **a** Schematic view of the honey bee brain with neurons important for olfactory processing. For simplification, neurons are shown only on one side of the brain. The *boxed area* shows the right antennal lobe (AL), which was imaged in all experiments. Olfactory input comes from the antenna via olfactory receptor neurons (ORN). Local neurons (*yellow*, *orange*) branch within the AL and are predominantly GABAergic. Multiglomerular projection neurons (PNs, *blue*) are also GABAergic and project to the lateral protocerebrum including the lateral horn (LH). Uniglomerular PNs (*green*) lead from the AL to the mushroom bodies (MB) via the lateral and medial antenno-protocerebral tract (lAPT, mAPT). Dye was injected into PNs between the median and the lateral calyx (*black cross*, mC, lC) of the MB. MB intrinsic cells are Kenyon cells (KC, *purple*), with cell bodies in and around the calyces and axons descending into the α-lobe, γ-lobe and β-lobe (αL, γL, βL). The unpaired octopaminergic VUM neurons (*red*) have their soma in the subesophageal ganglion (SEG) and innervate the ALs, the LHs, and the MB calyces. **b** Experimental procedure: odor responses were measured in sequence, followed by long background activity measurements. *Different colored boxes* represent different odors or dilutions. Note that 1-nonanol at 1:100 was measured twice. Four blocks of odor pulses were measured in total: before treatment, with 1 mM octopamine (OA), with 10 mM OA, and wash. **c** Example for a spatial odor response (*false-color coded*, see *color sequence right*), with overlaid glomerular borders. Upon odor stimulation (*gray bar*, nonanol), the calcium concentration increases in some glomeruli (*red*, e.g. A17, A33), and decreases in others (*dark blue*, e.g. A29). **d** *Left* Example time traces for a 1-nonanol response. Glomeruli A17 (*red*) and A33 (*black*) both respond, but with different time courses. Response magnitude is in the range of 10 % fluorescence ratio change. Glomerulus 29 (*cyan*) is inhibited. Note that calcium decreases are always small in size, because resting calcium levels are already low. *Right* Background activity in the same glomeruli. Note the small amplitude (different *y* axis scale) as compared to an odor response. Different glomeruli are not correlated over long time stretches

We stained PNs of the right antennal lobe (AL) by backfilling them with the calcium-sensitive dye Fura2-dextran (potassium salt, 10,000 MW, Invitrogen) as described elsewhere (Sachse and Galizia 2003; Galizia and Vetter 2004; Locatelli et al. 2013). A window was cut in the head capsule, and medial and lateral mushroom body calyces were exposed by carefully removing glands and tracheae. We inserted the tip of a glass electrode covered with Fura2-dextran into the protocerebrum slightly ventral to the place where medial and lateral calyx meet (Fig. 1a), aiming at axon bundles of the antenno-protocerebral tracts (lAPT and mAPT) that contain axons of uniglomerular projection neurons (Abel et al. 2001; Galizia and Rössler 2010). After the dye bolus dissolved inside the tissue, we removed the glass electrode and rinsed the brain with saline solution to remove excess dye from the brain surface. The head capsule was then closed and sealed with eicosane (Sigma-Aldrich) using the piece of cuticle that had been removed. The dye was allowed to travel along the axons for several hours.

Before imaging, we fixed the antennae at their bases to the head capsule with eicosane. Using fine forceps, the esophagus and supporting chitin structures were carefully pulled outwards through a hole in the cuticle and kept under tension to prevent esophageal movement. Then we reopened the window between the antennae and the ocelli and rinsed the brain with saline. Glands and trachea covering the right AL were pushed aside or, if necessary, removed to get visual access to the AL.

Calcium imaging was done using a CCD camera (image size 130 × 140 pixels; SensiCamQE, T.I.L.L. Photonics, Gräfelfing, Germany) mounted on an upright fluorescence microscope (Olympus BX-50WI, Japan) equipped with a 20 × dip objective, NA = 0.95 (Olympus), 505 nm dichroic mirror, and 515 nm LP filter (T.I.L.L. Photonics). Monochromatic excitation light (PolichromeV, T.I.L.L. Photonics) alternated between 340 and 380 nm. Exposure times differed between 5 and 15 ms for the 380 nm excitation light, depending on how intensely the AL was stained. For 340 nm, the exposure time was set 4 times longer than for 380 nm. Double images were taken at a sampling rate of 4 Hz for background activity and 5 Hz for odor responses. Image acquisition and light exposure were controlled by TILLVision software (T.I.L.L. Photonics).

### Odorant stimulation

We recorded the responses to the odorants 1-nonanol, 1-hexanol and 2-heptanone (all odorants from TCI, America). These odorants have been used in several previous studies, and are well characterized both behaviorally and physiologically, in particular with respect to which olfactory glomeruli they activate (Galizia et al. 1999b; Sachse et al. 1999; Guerrieri et al. 2005). Odorants were diluted 1:100 in mineral oil (Sigma-Aldrich). In addition, we recorded a concentration series for 1-nonanol, with concentration steps at 10^−4^, 10^−3^ and 10^−2^ dilution. Thus, 1-nonanol 1:100 was recorded two times in each set. 5 μl of odorant solution was loaded on a 0.5 × 4 cm filter paper strip and placed in a 1 ml glass syringe. The glass syringes were placed in a custom made olfactometer. A charcoal filtered air stream (25 ml/s) continuously flowed through the olfactometer and ventilated the antennae. A three-way valve (LFAA1200118H; The LEE Company) 4 cm upstream from the odorant cartridge controlled the onset of the airflow through the odorant cartridge. Valve opening (stimulus length: 2 s) was synchronized with the optical recordings directly from the TILLVision software. When the valve was open, the odorant-laden air from a cartridge was pushed into the continuous air stream in a mixing chamber. Odorant delivery was targeted at the antenna and positioned 1 cm away from the bee’s head. An exhaust placed 5 cm behind the bee continuously removed air from the arena.

### Drugs and solutions

All imaging experiments were performed under saline solution containing (in mM) 130 NaCl, 6 KCl, 4 MgCl_2_, 5 CaCl_2_, 160 sucrose, 25 glucose, 10 HEPES; pH 6.7, 500 mOsmol (all chemicals from Sigma-Aldrich). dl-Octopamine HCl (Sigma-Aldrich) was dissolved in saline solution to final concentrations of 1 and 10 mM of octopamine. Osmolarity was adjusted by reducing the amount of sucrose in the saline.

### RNA interference

dsRNA was synthesized following the PCR template method (Kennerdell and Carthew 1998) using T7 RNAP promotor linked oligonucleotides. PCR primers for *Amoa1* were: TAATACGACTCACTATAGGGAGACCACGAGACGAAGGCGGCGAAGACAC and TAATACGACTCACTATAGGGAGACCACCGTTTGCAGAAGCACTTGACGATG. This sequence produced a 294 bp DNA fragment that was then used as the template for dsRNA production. As a control, dsRNA was also synthesized corresponding to the *Drosophila fred* gene as disruption of *fred* has been shown to have significant effects in *Drosophila* (Chandra et al. 2003). This construct does not contain the level of sequence homology necessary to induce RNA interference in honey bee. PCR Primers used to produce the 822 bp DNA *Drosophila fred* (*Dmfred*) template were: TAATACGACTCACTATAGGGAGACCACATGGTGACATTGGAAATACACAG and TAATACGACTCACTATAGGGAGACCACCCTCTTATGCTGTCCAAAGGAT. dsRNA was synthesized in vitro from the PCR templates using the Maxiscript kit (Ambion), ethanol precipitated, resuspended in injection buffer (5 mM KCl; 10 mM NaH_2_PO_4_, pH 7.8), quantitated and diluted to 125 ng/μl in injection buffer.

Bees were prepared and their ALs exposed as described above. For brains to be used for calcium imaging, 4 nL of buffer containing 125 ng/μl of either *Amoa1* or *Dmfred* dsRNA was injected into the right AL using a picospritzer (Parker Hannifin Corporation). After injection, bees to be used in calcium imaging had their PNs of the right AL backfilled as described above. In a previous study, a significant reduction in receptor expression had been found 24 h after injection of dsRNA (Farooqui et al. 2003). Therefore, calcium imaging and dissection of ALs for western analysis were done 24 h after the animal had been injected with the dsRNA.

### Western analysis

To show that injection of *Amoa1* dsRNA, but not control dsRNA, lead to downregulation of the AmOA1 receptor, western analysis was used to quantitate AmOA1 protein levels in the ALs of bees injected with *Amoa1* dsRNA, control (*Dmfred*) dsRNA and bees that had their ALs exposed during surgery, but did not have any dsRNA injected. 24 h after injection with dsRNA or surgery alone, honey bee ALs were homogenized in 2× sample buffer (0.125 M Tris, 4 % SDS, 20 % glycerol, 0.2 mM DTT, pH 6.8), immediately boiled for 3 min, and stored at −70 °C. Homogenate was separated on a 7.5 % acrylamide Tris–glycine gel. Proteins were transferred onto nitrocellulose membrane (Bio-Rad Laboratories, Inc., Hercules, CA, USA) in transfer buffer (25 mM Tris, 192 mM glycine, 15 % methanol) at 0.45 A for 2 h at 4 °C. The membrane was blocked overnight in TBSTw (10 mM Tris, pH 7.5; 30 mM NaCl; 0.1 % Tween-20) plus 10 % low fat powdered milk at 4 °C and incubated in AmOA1 antiserum at 1:2,000 in TBSTw plus 2.5 % milk for 4 h at room temperature. Polyclonal antibodies against AmOA1 were generated against a 15 amino acid peptide (NH_2_-DFRFAFKSIICKCFC-OH) in the carboxyl terminus of the receptor and have been characterized previously (Farooqui et al. 2003; Sinakevitch et al. 2011). Following four 15 min washes in TBSTw plus 10 % milk, the membrane was incubated in anti-rabbit IgG HRP-conjugated secondary antibodies (Rockland Inc.) at 1:10,000 in TBSTw plus 2.5 % milk for 2 h. The membrane was washed three times in TBSTw with the final wash in TBS with no tween, and then developed using chemiluminescence as described by the manufacturer (Immobilon Western Chemiluminescent HRP Substrate; Millipore Corporation). As a loading control, after blotting with AmOA1 antibodies, the membrane was re-probed with anti-tubulin antibodies (Abcam Inc., Cambridge, MA, USA) at 1:10,000 and processed as above. The tubulin antibody produced a band at approximately 52 kDa. Images for quantification were captured using a ChemiDoc XRS gel imaging system (Bio-Rad Laboratories, Inc., Hercules, CA) and analyzed using ImageJ version 1.41o (Wayne Rasband, National Institutes of Health, USA, http://rsb.info.nih.gov/ij).

### Data analysis

We identified glomerular borders in calcium-imaging recordings based on their individual temporal dynamics, both in spontaneous background activity and in odor responses (Strauch and Galizia 2008). This approach uses a combination of principal component analysis (PCA) and Independent Component Analysis (ICA). First, we corrected for animal movement by aligning consecutive images to each other. This was achieved by performing a locally restricted cross-correlation on edge-enhanced thumbnail images. We used *z*-scores to normalize each of the 130 × 140 time series, i.e. we subtracted the mean and divided by the standard deviation. *Z*-score normalization was performed individually for each individual odorant stimulation or spontaneous activity measurement. Next, we performed dimensionality reduction with a covariance-free PCA algorithm (Papadimitriou et al. 2005), which avoided the construction of huge covariance matrices that would normally arise when applying conventional PCA to imaging datasets. Then, we applied the ICA algorithm fastICA (Hyvarinen and Oja 2000) to a complete recording (all measurements for the animal). The ICA approach resulted in spatially local and contiguous components, i.e. numerous objects consisting of time series that are mutually correlated with each other but uncorrelated to the time series in the other objects. We identified these spatial components as glomeruli when they were globular and within the anatomical boundaries of the AL and/or showed a response to odorant stimulation. Based on anatomical position of the glomeruli and their response dynamics to the tested odorants, we could assign names to glomeruli using the AL standard atlas (Flanagan and Mercer 1989; Galizia et al. 1999a). Glomerulus nomenclature was abbreviated for simplification: glomeruli innervated by the T1-tract were named with an A as prefix (e.g. T1-17 and T1-33 as A17, A33), while glomeruli innervated by the T3-tract were named with a C as prefix (e.g. T3-45 as C45), as done elsewhere (Galizia et al. 1999a). All the above processing (except for the movement correction) was only applied to detect and identify glomeruli. Once glomeruli were identified, we extracted glomerular time series from the movement-corrected but otherwise untreated imaging data. We extracted time series from the center of the identified glomeruli and averaged over a radius of 5 pixels to reduce local photon shot noise. We implemented all the above methods in Java.

Average calcium level is related to the absolute ratio level in FURA recordings: here we took the untreated ratio *F*_340_/*F*_380_. Other time-traces were normalized to pre-stimulus magnitude, and computed as log-fold change: $x_{i} : = { \ln }\left( {\frac{{F(340)_{i} /F(380)_{i} }}{{{\text{mean}}({\text{prestimulus}})}}} \right),$ with $x_{i}$ being the resulting signal measure for each timepoint *i*, and “prestimulus” being all 30 frames before stimulus, i.e. ${\text{mean}}({\text{prestimulus}}) = \frac{1}{30}\mathop \sum\nolimits_{i = 0}^{29} F(340)_{i} /F(380)_{i}$.

In order to evaluate changes in background activity, we calculated the standard deviation of the fluorescence ratio (*F*_340_/*F*_380_) for each glomerulus in all bees. An increase in standard deviation indicates an increase in number and amplitude of calcium fluctuations, which is typical for an increased background activity.

Odor stimulation can either increase or decrease intracellular calcium concentration, indicated by an increase or decrease in fluorescence ratio. As a measure for response strength, we calculated the response maximum and the mean fluorescence ratio within a 3 s time-interval (for glomeruli that showed calcium decreases to an odor we calculated a negative maximum, i.e. the minimum).

### Experimental procedure

Staining intensity, background activity and strength of an odor response can differ across individuals. We therefore followed a within-animal approach: each bee was first challenged with three odorants, and one nonanol concentration series, which were recorded with 5 Hz time resolution. Then, background calcium activity was measured for 125 s with 4 Hz time resolution. Next, and always in the same sequence, the same measurements were repeated with superfusion of 1 mM OA, 10 mM OA, and washing the brain with saline solution (Fig. 1b). Statistical data analysis was done by use of repeated measurements ANOVA, which takes into account that more than one set of recordings was performed on each animal. We used the programs SigmaStat and SigmaPlot (SPSS, IBM, USA) or the statistical language R (http://www.r-project.org/).

## Results

Odorant stimulation elicits an odor-specific pattern of glomerular activation in the antennal lobe (AL) of the honey bee (Joerges et al. 1997; Galizia et al. 1999b). We recorded the glomerular response to the odorants 1-hexanol (10^−2^), 2-heptanone (10^−2^) and 1-nonanol (concentration series 10^−4^, 10^−3^ and 10^−2^). Upon stimulation with an odorant, a characteristic glomerular response pattern was visible across the AL (Fig. 1c), with some glomeruli increasing, and some decreasing calcium concentration (Fig. 1d). Calcium increases were generally steep, with a slower decay phase after stimulus offset. Odor-response time courses differed for different glomeruli, some with a slow decay, some with a faster decay (Fig. 1c, d). The decrease of intracellular calcium concentration in some glomeruli was most likely due to a closing of calcium channels, while calcium pumps were still active. Given that resting calcium levels, which we measure as average calcium level, are generally low in neurons, the absolute values of calcium decrease were not high, as visible from the small downward deflection in the respective traces (Fig. 1d). When no odorant was given, glomeruli had constantly fluctuating background calcium concentration levels (Fig. 1e). These fluctuations were much smaller than odor responses (compare the ordinate axis in Fig. 1d and e), and the correlation across glomeruli was low, as previously reported (Galan et al. 2006).

### Octopamine increases background activity in projection neurons

**Fig. 2:**
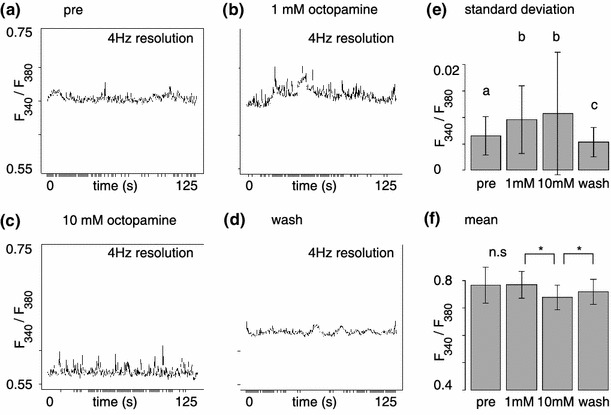
Octopamine increases background activity. Representative background activity time trace of a single glomerulus before treatment (pre, **a**), with 1 mM OA (**b**), with 10 mM OA (**c**) and after treatment (wash, **d**). Note the increased background activity in *b*, and the decreased mean but increased fluctuation in *c*. During wash activity (both mean value and fluctuations) returned to pre-treatment levels. **e** Aggregate statistics of the standard deviation in the signal (207 glomeruli from 13 animals followed over four stages): the OA-dependent increase in background activity is statistically significant (Friedman-test, SD by treatment: *p* < 2.2 × 10^−16^, stratified by animal). **f** Aggregate statistics of the mean fluorescence ratio. There was a significant drop at 10 mM OA that did not recover entirely in the wash (207 glomeruli from 13 animals followed over four stages, Friedman-Test, mean by treatment *p* < 2.2 × 10^−16^, stratified by animal). **e**, **f** Show mean and SD

Superfusing the brain with OA solution led to changes in background activity. Calcium fluctuations increased both with 1 mM OA (Fig. 2b) and with 10 mM OA (Fig. 2c). These fluctuations came back to baseline after OA was washed out. Furthermore, overall calcium levels dropped, in particular with 10 mM OA, as visible in Fig. 2c by the lower level of the curve. Across animals, the increase in calcium fluctuations was significant for 1 mM OA and for 10 mM OA (Fig. 2e), while the drop in overall calcium levels was only significant for 10 mM OA (Fig. 2f). The simultaneous increase in background activity and decrease in calcium levels (e.g. Fig. 2c) indicate that changing background activity in projection neurons is not caused by intrinsic mechanisms of the PNs themselves, but rather by synaptic input from the AL network.

### Octopamine modulates the odor response by inducing variable changes in glomerular activity

**Fig. 3:**
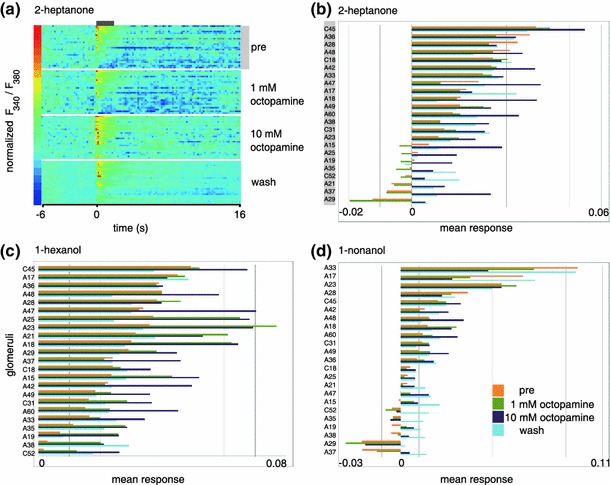
Octopamine modulates odor responses up and down. **a** False-color coded odor-response traces over time (time from left to right, odor 2-heptanone, one animal). Each line is one glomerulus; glomeruli are sorted by their response strength before treatment (“pre”, same order as in *b*). Note that some glomeruli have longer responses than others, some have long-lasting inhibitory responses, and some have off-responses (i.e. calcium increase at odor-offset; these have generally weak odor-on responses). **b**, **c**, **d** Glomerular response strength to 2-heptanone (**b**), 1-hexanol (**c**), 1-nonanol (**d**) in the pre, 1 mM OA, 10 mM OA and wash conditions (one animal). For each plot, glomeruli are arranged according to response strength in the pre-condition. Note that most glomeruli increase their response to odor in the presence of OA (*blue bars*), and that negative responses are rare. However, some glomeruli decrease their response with OA (e.g. A28 to heptanone, A33 to nonanol). Compare with suppl. Fig. S1

**Fig. 4:**
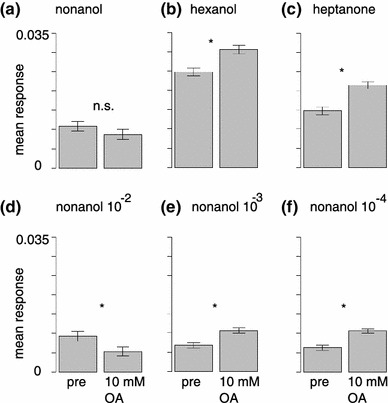
The octopamine effect is response-pattern specific. **a** Averaged across all glomeruli, responses to the first nonanol stimulus (10^−2^) did not decrease significantly after 10 mM OA application [Wilcoxon/Mann–Whitney with Holm correction for 6 tests (**a**–**f**), *p* = 0.34, 207 glomeruli from 13 animals, mean and SEM]. **b**, **c** Responses to hexanol (**b**) and heptanone (**c**) increased significantly (*p* = 5 × 10^−8^ and *p* = 2 × 10^−12^, respectively). **d** In the nonanol repetition (10^−2^) responses decreased (*p* = 3 × 10^−5^). **e**, **f** Responses to low-intensity nonanol stimulation increased significantly (*p* = 1 × 10^−10^ and *p* = 5 × 10^−13^, respectively, for odor concentrations 10^−3^ and 10^−4^)

Odors elicit complex patterns of calcium increase and decrease across glomeruli (Figs. 1c, 3a). The most characteristic property for each glomerulus is the response magnitude. Therefore, we quantified response magnitude across odors and glomeruli, and across identified glomeruli in different animals. We found that applying 1 mM OA modified the response patterns: some glomeruli increased their response, whereas other glomeruli decreased the response. Similarly, response magnitude changed after application of 10 mM OA. For example, glomerulus C45 increased its odor response considerably to 2-heptanone and to 1-hexanol (Fig. 3b, c). This was the strongest glomerulus for these two odors. However, glomerulus A33, which was the strongest glomerulus in the 1-nonanol response pattern, decreased its response to 1-nonanol after application of OA. Thus, the strongest glomeruli increased their responses for some odors, and decreased their responses for other odors. The effect on each glomerulus was odor-specific. For example, responses in glomerulus A17 decreased with OA treatment when 1-nonanol was given, but did not change for 1-hexanol (Fig. 3c, d). Indeed, the OA effect was not only odor-specific, but also concentration-dependent. For example, A17 increased its response to 1-nonanol at an odor concentration of 10^−4^, but decreased its response to the same odor at 10^−2^ (suppl. Fig. S1). A two-way ANOVA with the factors treatment and odors (with the different concentrations of 1-nonanol treated as different odors) found significant differences for the levels of treatment (*F* = 28.4, *p* < 0.001, i.e. OA had an effect) as well as for the levels of odor (*F* = 255.1, *p* < 0.001, i.e. different odors elicit different response patterns). Importantly, however, we found a significant interaction between odor and treatment (*F* = 4.2, *p* < 0.001), indicating that octopamine does not affect the response to all odors the same way. With OA superfusion the responses increased to the odors 1-hexanol and 2-heptanone as well as to 1-nonanol at concentrations of 10^−3^ and 10^−4^, but the responses to 1-nonanol 10^−2^ were either not affected (first stimulation) or reduced (second stimulation) in the presence of 10 mM OA (Fig. 4). The reduced effect with the first nonanol stimulation might be caused by the shorter time that octopamine had been present in the bath during this stimulation.

These observations indicate that increase or decrease in projection neuron responses within a glomerulus after OA treatment is not a property of that glomerulus, but rather a property of the AL network. The most prominent effect was that purely negative odor responses only rarely occurred under OA treatment: almost all negative responses reverted to positive ones under different treatment conditions.

### The effect of octopamine depends on the strength of the initial odor response

**Fig. 5:**
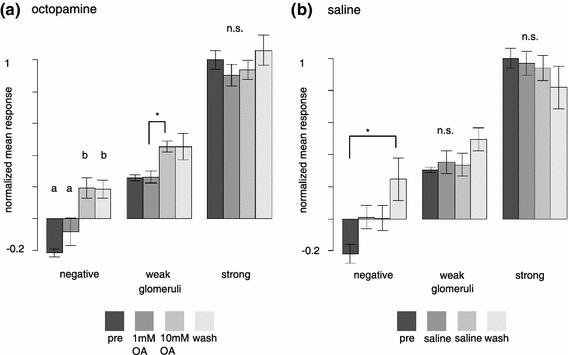
The octopamine effect is related to odor-response strength. **a** Octopamine converts odor responses from negative to positive, and increases weak odor responses. Strong odor responses are not increased when pooled across all strong odor responses. All responses are scaled to the mean pre-treatment response in strong glomeruli (set to 1). Mean and SEM. See “Results” for statistical tests. **b** Without OA treatment there is also a tendency for negative responses to become positive with repeated odor stimulation over time. This effect is slower than with OA treatment, i.e. it becomes visible only in the fourth measurement block (“wash”). There is no significant change for positive odor responses, irrespective of whether weak or strong. Responses were scaled as in *a*. Mean and SEM. See “Results” for statistical tests

We noted that strong odor responses were less likely to be modulated by OA treatment than weak odor responses. To test this, we created three groups of glomeruli based on their response strength to each particular stimulus: negative, weak, and strong (39 classified responses from 13 bees, *N* = 13 for each group). We chose 2.5 × mean standard deviation of the background activity, measured during 6 s before odorant onset, as a threshold. Glomeruli with a mean odor response that was below threshold before octopamine application were defined as weak glomeruli, glomeruli with an initial response above threshold as strong glomeruli. Glomeruli responding with a decrease in calcium concentration formed the “negative” group. Across all odors, concentrations and animals, the increased response for weak responses and for intermediate responses was significant, but no significant effect was visible for high responses (Fig. 5a). Specifically, inhibited glomeruli not only showed less negative responses, but generally even positive responses to odorant stimuli when 10 mM of octopamine was applied (e.g. A21 and A37 in Fig. 3b), an effect that was highly significant (Tukey HD following Friedman repeated measures ANOVA, χ^2^ = 23.7, *p* < 0.001). The effect was not reversible within 10 min. The observed switch from calcium decrease to calcium increase in the odor responses suggests a strong decrease in inhibitory input to a glomerulus. Weak glomeruli significantly increased their odor responses after application of 10 mM octopamine (Fig. 5a, Tukey HSD following Friedman repeated measures ANOVA, χ^2^ = 18.5, *p* < 0.001). In strong glomeruli we observed no clear increase in mean odor response but rather a tendency to decrease the odor response in the presence of OA (Fig. 5a). However, this decrease in mean odor response was not statistically significant (one-way ANOVA, *F* = 1.277, *p* = 0.314).

Given that in the control situation, when the entire experiment was done with saline treatment rather than OA treatment, the negative glomerular responses also became positive, albeit after a longer time than in the OA case (Fig. 5b, 23 classified responses from 8 bees), it appears that, at least for the calcium-decrease case, there may be two (or more) overlapping mechanisms: a reduction of negative responses over long measurement times with repeated stimulation and recording (Fig. 5b), and an immediate decrease due to OA treatment (Fig. 5a).

### The effect of octopamine is variable across animals

For further analysis, we performed a two-way ANOVA with the factors treatment (with or without OA) and glomerulus (glomerular identity) and found significant differences for both factors, but no interaction between factors (two-way ANOVA, *p*_treatment_ = 1.1e−08; *p*_glomerulus_ < 2.2e−16; *p*_interaction_ = 1). These results again show that octopamine treatment led to a significant difference in mean odor response. Additionally, it shows that the measured glomeruli differed significantly in their mean odor responses, which is not unexpected as all glomeruli have an individual odor-response profile. However, the lack of interaction between factors strengthens the hypothesis that glomerular identity did not determine the response to OA treatment. Moreover, when pooling individual glomeruli across animals, we found significant octopamine-induced changes in mean odor response across odors only in two glomeruli, namely in glomeruli 37 and 49 (Tukey HSD following Friedman repeated measures ANOVA, χ_37_^2^ = 14.8, *p*_37_ = 0.002 χ_49_^2^ = 20.6, *p*_49_ < 0.001; to adjust for multiple testing, significance level was corrected by use of Bonferroni-correction; compare with suppl. Fig. S2). Thus, most glomeruli could both increase or decrease activity in the presence of octopamine.

### The octopamine effect is a network effect

**Fig. 6:**
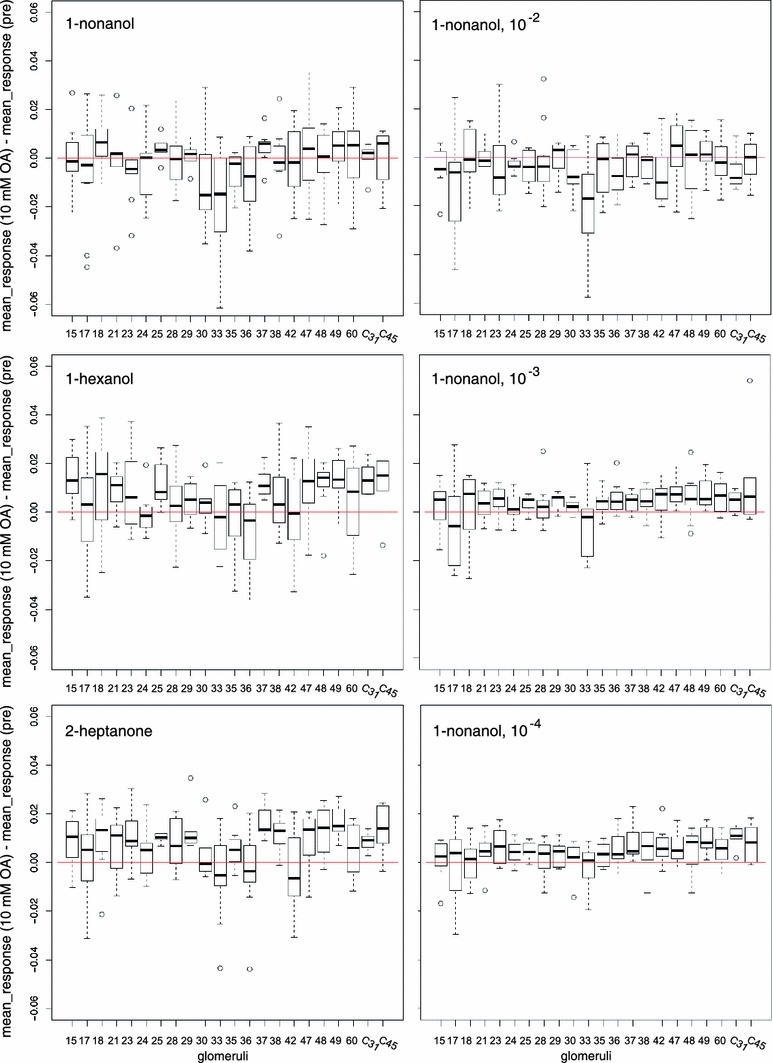
The octopamine effect is variable across animals. The OA-mediated change in odor response (OA treatment response *minus* pretreatment response) resolved for different odors: 1-nonanol, 1-hexanol, 2-heptanone and different concentrations: 1-nonanol (separate measurement set), 10^−2^, 10^−3^ and 10^−4^. Note that for all odors, OA-induced changes vary widely, with both positive and negative effects. This indicates that there is a high variability across animals, suggesting a role of individual network plasticity. *Box plot* with mean and quartiles, *whiskers* indicate the range (min–max or 1.5 × interquartile distance, whichever smaller), *circles* are values outside this range (outliers). For all odors, we show the same 189 glomeruli. Number of animals differs for glomerulus: mean = 8.59, SD = 2.72, required minimum for analysis was *n* = 5 animals

Next, we investigated whether glomeruli had stereotypical responses to OA treatment. Figure 6 shows bar-plots of odor-response differences for each odor. What is apparent is that the variability is high. Importantly, not even the polarity is uniform. For example, glomerulus A30 showed all ranges of increases and decreases of responses to 1-nonanol after OA treatment, as did glomerulus A18 to 1-hexanol, or glomerulus A33 to 2-heptanone. Weak and strong glomeruli were equally variable (e.g. A33 to 1-nonanol as a strong glomerulus). This variability indicates that OA may act on a network that is not innate, but rather the result of plasticity and/or genetic variability, and thus variable across animals.

OA acts on receptors that increase intracellular calcium concentration. Since we found both response increases and response decreases, it is unlikely that receptors on PNs contribute significantly to the effects shown here. In order to test this explicitly, we superfused the brain with caffeine, which leads to a general increase in intracellular calcium. This treatment led to a general increase in odor responses (at 5 mM caffeine), or a general decrease in odor responses (at 20 mM caffeine), but never to a complex pattern of increases and decreases in different glomeruli (suppl. Fig. S3). Thus, the OA effect reported here does not result from a general intracellular calcium increase, but rather must be a network-specific effect.

### The role of AmOA1

**Fig. 7:**
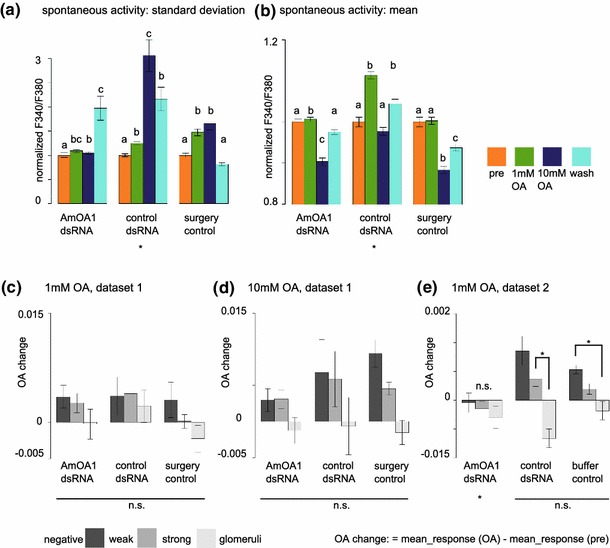
Downregulating AmOA1 octopamine receptors abolishes the octopamine effect. **a** With control dsRNA injection, and in the non-injected control (surgery), the increase in spontaneous activity was more pronounced than when AmOA1 receptor levels were downregulated by dsRNA injection. Data are normalized to pre-OA values (set to 1). Mean and SEM. See “Results” for statistical tests. **b** Background calcium levels were not affected in a systematic way. Mean and SEM. See “Results” for statistical tests. **c**, **d** When glomeruli are grouped as giving negative, weak or strong responses (compare with Fig. 5), odor-induced responses in negative and weak signals were higher for control animals than for *Amoa1* dsRNA injected animals, although this effect was not significant due to high variability in odor-response strength (note the error bars). Mean and SEM. See “Results” for statistical tests. **e** In an independent dataset, OA had a highly significant effect in control animals, which was abolished by *Amoa1* dsRNA treatment. Buffer control animals were injected with injection buffer only, and no dsRNA. Mean and SEM. See “Results” for statistical tests

All results so far indicated that OA acts as a modulator within the AL. However, other explanations are also possible. In particular, OA could have nonspecific effects on other biogenic amine receptors, e.g. tyramine receptors, which share a high sequence similarity. Furthermore, OA may act in brain areas other than the ALs, and the effects seen here might be mediated by neural feedback connections into the AL. In order to elucidate whether the observed effects indeed originate within the AL, and are caused by OA receptors, we downregulated the expression of the OA receptor AmOA1 using RNA interference. We injected either *Amoa1* or control (*Dmfred*) dsRNA, in addition, a third group of animals either underwent surgery but was not injected (surgery, Fig. 7a–d) or was injected with the injection buffer used to dilute the dsRNA (Fig. 7e). We injected the dsRNA into the right ALs, and 24 h later recorded spontaneous activity and odor responses. At the end of the experiment, ALs were dissected and used for western blot analysis. Injection of *Amoa1*, but not control dsRNA, led to a significant reduction in AmOA1 receptor protein levels (suppl. Fig. S4).

Background activity increased in control dsRNA animals in the presence of octopamine (Tukey HSD following Friedman repeated measurement ANOVA χ^2^ = 84.305 *p* < 0.001, Fig. 7a; AmOA1 dsRNA: 192 glomeruli from 13 bees; surgery control: *n* = 207 from 13 bees, control dsRNA: *n* = 169 from 10 bees). However, animals that had been treated with *Amoa1* dsRNA did not show an increase in background activity in presence of octopamine, but there was a significant increase in background activity after the washout. Background calcium levels (i.e. mean spontaneous activity) were not affected by injection of either *Amoa1* or control dsRNA (Fig. 7b). Sorting glomeruli by response strength (compare with Fig. 5) confirmed a strong increase in responses, in particular for inhibited and weak glomeruli, in the untreated (surgery or injection buffer) and in the control dsRNA treatment (Fig. 7c, d; AmOA1: 39 classified responses, 13 negative, 13 strong, 13 weak from 13 bees; control dsRNA: *n* = 30 responses from 10 animals; surgery: *n* = 39 from 13 animals. Same animals for 1 mM OA and for 10 mM OA. Figure 7e: AmOA1: 27 classified responses from 9 bees; control dsRNA: *n* = 30 responses from 10 animals; surgery: *n* = 26 from 9 animals). In contrast, after *Amoa1* dsRNA injection, there was only a weak and non-significant odor-response increase in inhibited and weak glomeruli. Thus, *Amoa1* dsRNA injection almost completely abolished the OA superfusion effect, indicating that AmOA1 receptors within the AL were responsible for the observed modulations provoked by OA.

## Discussion

Behavioral studies have shown that octopamine (OA) in the antennal lobe (AL) plays an important role in appetitive odor learning (Hammer and Menzel 1998; Farooqui et al. 2003). OA-like immunoreactivity has been detected in glomeruli and within the coarse central neuropil of the AL (Kreissl et al. 1994; Sinakevitch et al. 2005), and antibodies against the honey bee OA receptor AmOA1 stain different groups of AL neurons including a group of GABAergic local interneurons (Sinakevitch et al. 2011). We show here that the neurotransmitter OA modulates the neural networks in the honey bee AL. Most importantly, we show that the effect of applying OA is not uniform across all olfactory glomeruli. The effect differs across glomeruli and is likely related to network connectivity. The effect is dependent on several factors (most importantly odor, odor concentration, glomerulus), and it has a high variability across animals (Fig. 6). The inter-animal variability in particular suggests that modulation by OA is related to the individual life history of the animal or other factors such as genetic background, and that it plays a role in olfactory memory.

Specifically, we found that applying OA increases background activity of olfactory projection neurons (PNs), i.e. these neurons have a higher level of activity within the network even in the absence of olfactory stimulation (Fig. 2). Background activity in PNs is a network effect and driven, in part, by spontaneous activity in olfactory receptor neurons. The increased activity when applying OA is not due to an increase in the basal level of Ca^2+^ (Fig. 2f) which would lead to a lowered threshold of the PN itself. Rather, it is likely driven by external factors, e.g. by decreased inhibitory input (see model proposed below). This view is strengthened by our observation that odor responses are also modified by OA, but in a non-uniform way: some glomeruli increase while others decrease their odor response after OA treatment (Fig. 3). Even more intriguingly, some glomeruli increase their response to one odor, but decrease the response to another, or even to the same odor at another concentration (suppl. Fig. S1). Generally, glomeruli most likely to be modulated are those with negative or weak responses (Fig. 5), keeping in mind that not all negative or weak responses are modulated, and some strong glomeruli may be modulated for particular odors.

### OA acts on the antennal lobe network

Therefore, while the network effect of OA was reproducible for a given stimulus within a given animal, it was not predictable from one odor to another, or from one animal to another. This observation suggests that OA acts to a large degree at a network level on synaptic contacts, rather than on the excitability of particular cells. The latter was confirmed in a control experiment (suppl. Fig. S3): increasing intracellular calcium levels with caffeine led to a similar increase in background activity as OA, but odor responses were globally increased. This shows that when the increase in background activity is due to a general intracellular threshold shift, the network property revealed by OA is abolished. Similarly, treatments with drugs that inhibit GABA_A_- or histamine-receptors also lead to a nonspecific increase in background activity in projection neurons (Sachse and Galizia 2002; Sachse et al. 2006).

In our experiments we applied OA to the entire brain, begging the question of where the effect is localized: within the antennae on sensory neurons, as shown in cockroaches (Flecke and Stengl 2009), in some brain areas that have neural projections to the AL, or in the AL itself? When we downregulated AmOA1 receptors via localized injection of dsRNA into the AL, the result was a total abolishment of increased spontaneous activity (Fig. 7a), and a reduced effect in odor-response modulation in the presence of OA (Fig. 7c–e). Thus, at least part of the octopamine effect is likely mediated by AmOA1 receptors within the AL. Nevertheless, other OA receptors may be involved as the bee genome encodes OA receptors of the Octβ receptor class as well as AmOA1 (Evans and Maqueira 2005). Furthermore, feedback connections from other brain areas that need not themselves be octopaminergic may add to the effect, which remains to be tested. Importantly, we show a specific effect of an OA receptor, ruling out a nonspecific cross-reaction of the bath applied OA with other biogenic amine receptors.

### OA creates a filter for odor patterns

**Fig. 8:**
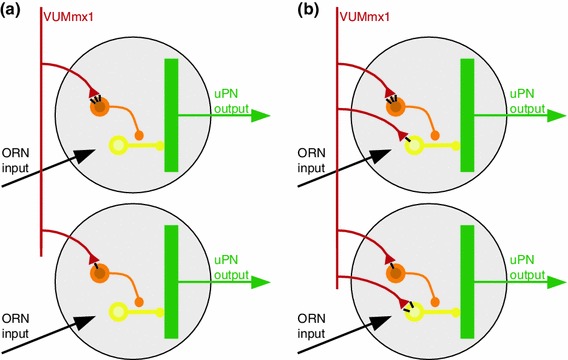
Putative model for effect of octopamine in the honey bee antennal lobe. **a** Our data and previously published data are consistent with the model shown (see text for details): Odor receptor neurons (ORN, *black*) are activated by the presence of an odor. Octopaminergic neurons (e.g., VUMmx1, *red*) make synaptic contacts with inhibitory local neurons (*orange*) which synapse onto other inhibitory local neurons (yellow) which synapse onto projection neurons (uPN, *green*, measured in this study). Synaptic strength (number of OA receptors, *black bars* in the Figure) differs in different glomeruli. Therefore, the effect of OA is quantitatively different from one glomerulus to the next, and not consistent across animals. When OA is present, the orange neuron is excited, thus the yellow neuron is inhibited, and as a consequence the projection neuron is disinhibited, i.e. its odor response is stronger. **b** A more complex model includes OA input onto local neurons that inhibit uPNs. With this addition, OA release (e.g. by VUMmx1 during appetitive training, or responding to a learned odor) will facilitate some glomeruli (via the circuit shown in **a**), and inhibit others (via the synapse onto the *yellow* neuron)

Since we see both up and downregulation of odor responses, the OA effect onto PNs must be mostly multisynaptic. We propose here that OA acts on a disinhibitory pathway within the AL (Christensen et al. 1993), specifically on a particular subset of GABAergic local neurons (LNs), which themselves act on other GABAergic neurons (Fig. 8a). With increasing OA, the OA target cells become more active, inhibiting their inhibitory synaptic partners, which leads to PNs being disinhibited, increasing their spontaneous activity. In this model, the synaptic efficiency of OA-to-LN synapses is plastic, and depends on previous experience. Thus, odor patterns that have previously been experienced in an appetitive context, that therefore led to an OA release, will be preferentially activated. In the natural environment, such a mechanism would ensure that the AL becomes more sensitive for odor-response patterns that have already successfully indicated the presence of food, and within these patterns the network becomes more selective for the common part of the pattern, i.e. for a consensus odor representation (Smith et al. 2006; Riffell et al. 2013).

This model (Fig. 8a) is based on the observations in this paper, combined with other previously published results. First, immunohistochemical analyses showed that AmOA1 receptors are localized on a subpopulation of GABAergic cells (Sinakevitch et al. 2011), although which specific GABAergic LN subpopulation expresses these receptor genes remains unknown. Also, we cannot exclude that in addition to GABAergic LNs, other neurons (e.g. histaminergic neurons or cholinergic neurons) might also respond to OA. In particular, two AL cell clusters label with antibodies against the AmOA1 receptor that do not co-label with an anti-GABA-antibody (Sinakevitch et al. 2011). Second, immunohistochemical analysis also shows that receptor density varies across glomeruli, and that this diversity is not predictable, i.e. if a glomerulus has a high OA receptor density in one animal, it may have a low density in another animal, supporting our suggestion that OA receptor density is experience dependent (Sinakevitch et al. 2011). Third, activation of AmOA1 receptors expressed in HEK cells leads to Ca^2+^ fluctuations and increased intracellular Ca^2+^ concentrations (Grohmann et al. 2003). In this study, we find an increase in Ca^2+^ fluctuations, but not in Ca^2+^ concentration, suggesting an indirect effect of OA onto PNs via the network, rather than a direct effect on PNs. Fourth, after appetitive learning, odor-response patterns change in the ALs of honey bees, but when averaging across individuals the effects are not reproducible, i.e. no change is visible (Peele et al. 2006)—again indicating a high network variability that may be experience dependent. Accordingly, when plasticity data is analyzed using multivariate techniques (Fernandez et al. 2009), or when glomeruli are sorted into odor-response classes (Rath et al. 2011), then clear shifts in odor-response profiles can be seen after appetitive olfactory conditioning, showing that network plasticity in the AL depends on previous experience.

Taking these results together, we propose that OA acts on a specific subpopulation of disinhibitory GABAergic LNs in the AL. It should be noted that the schematic in Fig. 8a does not depict the entire network within the AL, but only those elements that are affected by OA (for example, receptor neuron targets are not specified). Indeed, we are confident that further analyses will reveal more OA targets. For example, in several cases we did observe not an odor-response increase, but rather a decrease. The model in Fig. 8b illustrates one that would account for this observation: OA neurons could target both a disinhibitory and an inhibitory pathway onto PNs, and thus plasticity in receptor density would allow both for upregulation and downregulation of a glomerulus in the presence of an expected reward. The degrees of freedom that need to be tested increase in this model. For example, in Fig. 8b OA1 receptor density is approximately proportional for the disinhibitory and the inhibitory pathway—however, this is likely not to be the case, given the heterogeneous expression of OA1 receptors across glomeruli. Furthermore, in Fig. 8b both pathways share the same inhibitory neuron (yellow)—again, not a necessity. Indeed, the inhibitory pathway may not even be GABAergic, but use other inhibitory transmitters, such as histamine. Thus, the scenario of a parallel inhibitory and disinhibitory pathway modulated by OA offers many new hypotheses to be tested. Importantly, however, it offers the possibility that OA modulates glomeruli both up and down, and as a result an odor that activates VUMmx1 (because previously associated with a reward) will lead to a more reliable and stable representation in the brain.

It may well be that octopaminergic networks act in similar ways in other sensory systems in invertebrates as well. In crustaceans, OA either enhances or decreases transmitter release through neuromuscular junctions, and alterations in endogenous OA levels have been suggested to contribute to these variable responses (Djokaj et al. 2001). Daytime-dependent variations in OA levels in the hemolymph have also been suggested to alter the response to exogenously applied OA on pheromone-sensitive neurons in the hawkmoth *Manduca sexta* (Flecke and Stengl 2009). Finally, apparent or real variations in OA concentration might also cause variable physiological effects: frequency and force of heart contractions in isolated hearts of the honey bee are increased at high concentrations of OA, while low OA concentrations inhibit the heart firing rate (Papaefthimiou and Theophilidis 2011). In our case, these effects may be increased by differential OA receptor expression density.

The network properties underlying these models remain to be elucidated. More localized pharmacological analyses (Girardin et al. 2013) may help to disentangle the detailed network in the honey bee olfactory system. Computer models can provide insight if such a selective, disinhibitory modulatory system including experience-dependent plasticity can indeed create a selective filter for extracting common elements in an environment of fluctuating odorant stimuli, and thus help an animal to efficiently process and recognize odors. Importantly, our study indicates that the results of learning and plasticity may need to be analyzed at the level of each individual, because the network effects average out across individuals. Honey bees are ideal model animals to study these effects, given their robust learning rates and the ease of analyzing individually trained animals. In particular, it will be interesting to compare the effect of octopamine on naive and experienced bees, and thus examine the precise nature of how experience modifies the neural networks involved in olfactory processing.

## Electronic supplementary material

Below is the link to the electronic supplementary material.

**Supplemental Figure S1: effect of octopamine on responses to different concentrations of 1-nonanol** a) Responses to 1-nonanol at 1:100 dilution, plotted as in Fig. 3. Note that only few glomeruli are strongly active (notably A33, A23 and A17), and that several glomeruli are inhibited (dark blue traces at the bottom). Strong responses decrease and weak responses increase upon application of octopamine (see block for 10 mM octopamine). b) Response strength to 1-nonanol at 1:100 dilution, for identified glomeruli, comparing pretreatment (pre, orange), 1 mM OA (green), 10 mM OA (dark blue) and wash (pale blue). Note how strong responses decrease, intermediate responses remain unchanged, and negative responses increase. Also note that this relationship is not very strong: individual glomeruli can have changes different from their group. Glomeruli are sorted by pre response strength (see glomerular labels to the left). c) Same as *b*, for a lower intensity stimulation (nonanol 1:1,000). With 1 mM OA several inhibitory responses become visible that were not present before. Note the different abscissa scale. d) Same as *b*, *c*, for nonanol dilution 1:10,000. Note the different abscissa scale. Again, negative responses in most cases shift to positive responses, but some positive responses shift to negative responses. (PDF 187 kb)

Supplemental Figure S2: changes in glomerular odor responses after octopamine treatment The odor-response change (10 mM OA treatment response *minus* the pre response) resolved for identified glomeruli (abscissa) shows that change levels differ across glomeruli, but in most cases the median change is an increase in odor responses. Some glomeruli have very high variability, e.g. glomeruli A17 and A33, which form the core response pattern to 1-nonanol. This was also the odor with the most frequent odor-response decreases upon OA treatment. Boxplot with mean and quartiles, whiskers indicate the range (min**–**max or 1.5*interquartile distance, whichever smaller), circles are values outside this range (outliers). 189 glomeruli from 13 animals (same data as Fig. 6, pooled across odors). Number of animals differs for glomerulus: mean = 8.59, sd = 2.72, required minimum for analysis was *n* = 5 animals. (PDF 52 kb)

Supplemental Figure S3: caffeine modulates background activity a) Plot of odor responses before (abscissa) and after (ordinate) application of 1 mM OA (left) or 10 mM OA (right). Note that all values scatter widely around the diagonal, indicating that some glomeruli increase, while others decrease, their response upon OA application. 621 responses from 207 glomeruli, three odors, 13 animals. b) Plot of odor responses before (abscissa) and after (ordinate) application of 5 mM caffeine (left) shows that odor responses increase in most glomeruli (most points are left of the diagonal). 228 responses to three odors from 6 bees. With 20 mM caffeine treatment (right) odor responses decrease in most glomeruli (most points are right of the diagonal). There is no differential effect as for OA treatment, which produced both positive and negative modulation. 168 responses to three odors from 6 bees. 5 mM and 20 mM data measured in different bees. c) The mean spontaneous activity level without odor stimulation is related to the resting calcium level in the cells. Left: after application of 5 mM caffeine the calcium level decreases (left, *p* = 0.02, Wilcoxon signed rank test with continuity correction). Right: spontaneous activity (measured as standard deviation) increases in the presence of 5 mM caffeine (*p* = 0.04, Wilcoxon signed rank test with continuity correction, 77 glomeruli from 6 bees). (PDF 207 kb)

Supplemental Figure S4: *dsAmoa1* treatment reduces OA receptor density a) Example lanes from a western blot using an antibody against AmOA1 (top row) or tubulin (bottom row) for antennal lobes injected with control dsRNA (left), *Amoa1* dsRNA (middle), or bees undergoing surgery alone (right). b) Quantification of the level of AmOA1 protein relative to the amount of tubulin confirms a significant reduction in the level of AmOA1 in the ALs of bees treated with *Amoa1* dsRNA 24 h after injection (ANOVA, *p* = 0.046, n_surgery_ = 7, n_control dsRNA_ = 6, n_*Amoa1* dsRNA_ = 6) (PDF 122 kb)

**Supplemental movies: odor responses** Movies show odor responses under four conditions: before treatment (“pre”), during 1 mM OA, during 10 mM OA and after wash out (“wash”), as indicated in the file name. For example, “1-nonanol_10mM_OA.mp4” is the response to 1-nonanol during 10mM OA superfusion. (MP4 220 kb)

**Supplemental movies: spontaneous activity** Movies show spontaneous activity examples for the four phases: pretreatment, 1 mM OA, 10 mM OA and wash. (MP4 234 kb)

Supplementary material 7 (MP4 222 kb)

Supplementary material 8 (MP4 184 kb)

Supplementary material 9 (MP4 223 kb)

Supplementary material 10 (MP4 232 kb)

Supplementary material 11 (MP4 216 kb)

Supplementary material 12 (MP4 210 kb)

Supplementary material 13 (MP4 216 kb)

Supplementary material 14 (MP4 235 kb)

Supplementary material 15 (MP4 216 kb)

Supplementary material 16 (MP4 218 kb)

Supplementary material 17 (MP4 3724 kb)

Supplementary material 18 (MP4 662 kb)

## Abbreviations

AL: Antennal lobes
ANOVA: Analysis of variance
DsRNA: Double stranded RNA
HEK cells: Human embryonic kidney 293 cells
LN: Local neuron
MB: Mushroom bodies
OA: Octopamine
PCA: Principal component analysis
PCR: Polymerase chain reaction
PN: Projection neuron
VUM: Ventral unpaired median
