# Supplementary figures and images for: Octopamine modulates activity of neural networks in the honey bee antennal lobe
Source: J Comp Physiol A Neuroethol Sens Neural Behav Physiol. 2013 May 17;199(11):947–62. doi: 10.1007/s00359-013-0805-y (PMC3825135; doi:10.1007/s00359-013-0805-y)

a) 1-nanolol,  $10^{-2}$

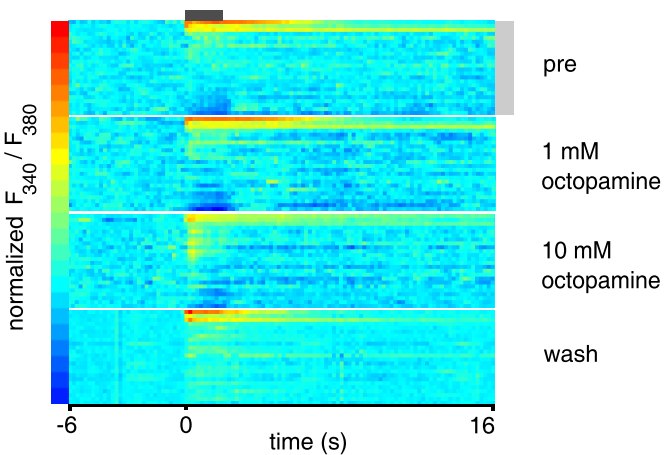

b) 1-nanolol,  $10^{-2}$

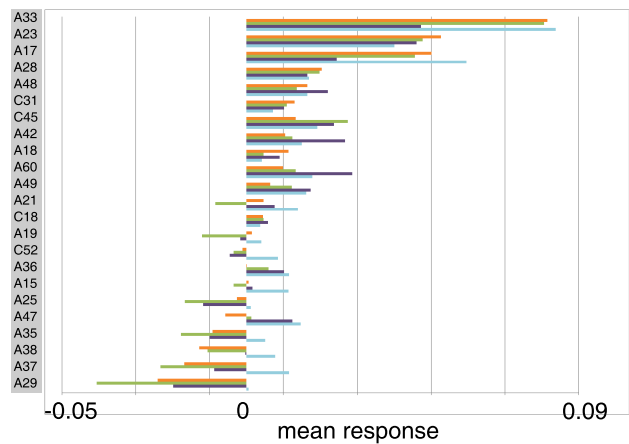

c) 1-nanolol,  $10^{-3}$

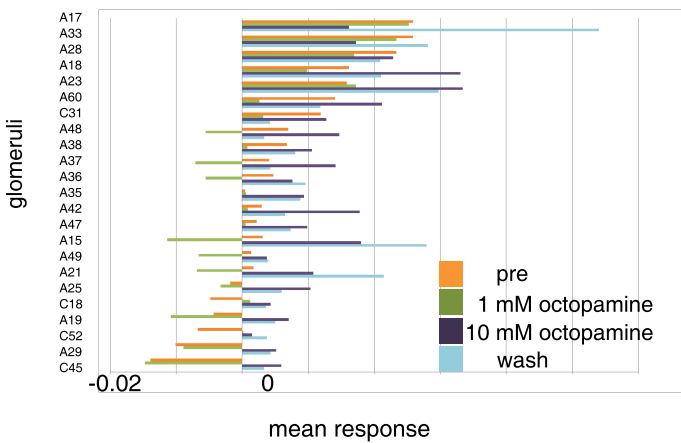

d) 1-nanolol,  $10^{-4}$

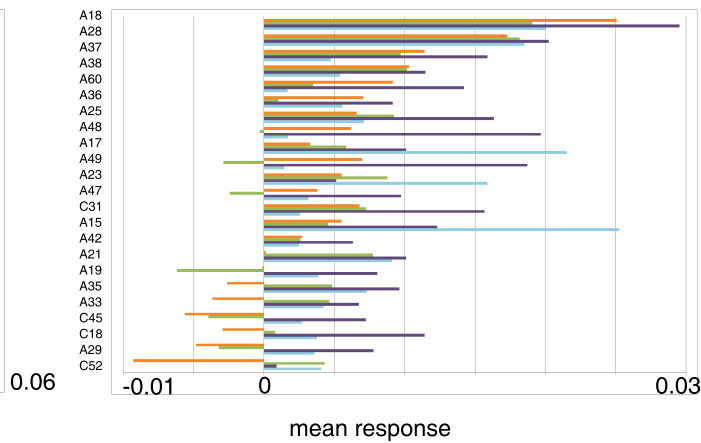

Supplement: Supplementary file 1 — Supplemental Figure S1: effect of octopamine on responses to different concentrations of 1-nonanol a) Responses to 1-nonanol at 1:100 dilution, plotted as in Fig. 3. Note that only few glomeruli are strongly active (notably A33, A23 and A17), and that several glomeruli are inhibited (dark blue traces at the bottom). Strong responses decrease and weak responses increase upon application of octopamine (see block for 10 mM octopamine). b) Response strength to 1-nonanol at 1:100 dilution, for identified glomeruli, comparing pretreatment (pre, orange), 1 mM OA (green), 10 mM OA (dark blue) and wash (pale blue). Note how strong responses decrease, intermediate responses remain unchanged, and negative responses increase. Also note that this relationship is not very strong: individual glomeruli can have changes different from their group. Glomeruli are sorted by pre response strength (see glomerular labels to the left). c) Same as b, for a lower intensity stimulation (nonanol 1:1,000). With 1 mM OA several inhibitory responses become visible that were not present before. Note the different abscissa scale. d) Same as b, c, for nonanol dilution 1:10,000. Note the different abscissa scale. Again, negative responses in most cases shift to positive responses, but some positive responses shift to negative responses. (PDF 187 kb) [file 359_2013_805_MOESM1_ESM.pdf]

mean\_response (10 mM OA) - mean\_response (pre)

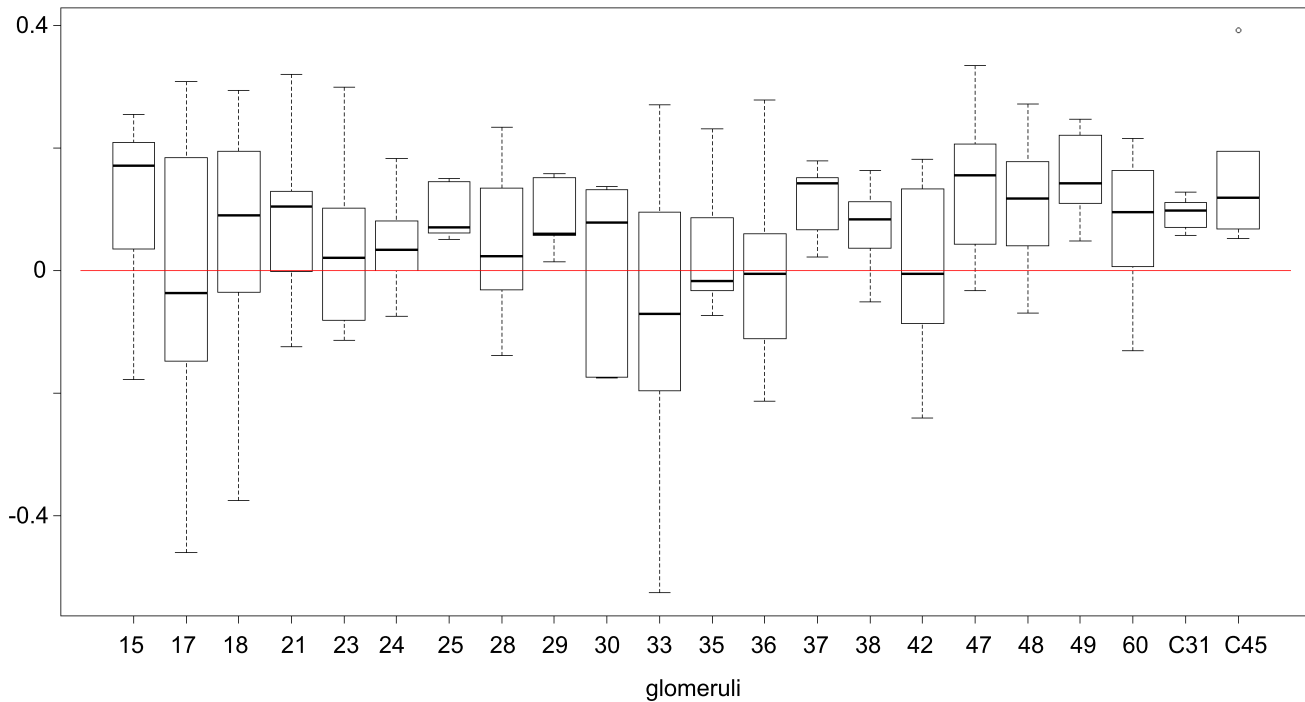

Supplement: Supplementary file 2 — Supplemental Figure S2: changes in glomerular odor responses after octopamine treatment The odor-response change (10 mM OA treatment response minus the pre response) resolved for identified glomeruli (abscissa) shows that change levels differ across glomeruli, but in most cases the median change is an increase in odor responses. Some glomeruli have very high variability, e.g. glomeruli A17 and A33, which form the core response pattern to 1-nonanol. This was also the odor with the most frequent odor-response decreases upon OA treatment. Boxplot with mean and quartiles, whiskers indicate the range (min–max or 1.5*interquartile distance, whichever smaller), circles are values outside this range (outliers). 189 glomeruli from 13 animals (same data as Fig. 6, pooled across odors). Number of animals differs for glomerulus: mean = 8.59, sd = 2.72, required minimum for analysis was n = 5 animals. (PDF 52 kb) [file 359_2013_805_MOESM2_ESM.pdf]
