# Supplementary material for: Octopamine modulates activity of neural networks in the honey bee antennal lobe
Source: J Comp Physiol A Neuroethol Sens Neural Behav Physiol. 2013 May 17;199(11):947–62. doi: 10.1007/s00359-013-0805-y (PMC3825135; doi:10.1007/s00359-013-0805-y)
Supplement: Supplementary file 3 — Supplemental Figure S3: caffeine modulates background activity a) Plot of odor responses before (abscissa) and after (ordinate) application of 1 mM OA (left) or 10 mM OA (right). Note that all values scatter widely around the diagonal, indicating that some glomeruli increase, while others decrease, their response upon OA application. 621 responses from 207 glomeruli, three odors, 13 animals. b) Plot of odor responses before (abscissa) and after (ordinate) application of 5 mM caffeine (left) shows that odor responses increase in most glomeruli (most points are left of the diagonal). 228 responses to three odors from 6 bees. With 20 mM caffeine treatment (right) odor responses decrease in most glomeruli (most points are right of the diagonal). There is no differential effect as for OA treatment, which produced both positive and negative modulation. 168 responses to three odors from 6 bees. 5 mM and 20 mM data measured in different bees. c) The mean spontaneous activity level without odor stimulation is related to the resting calcium level in the cells. Left: after application of 5 mM caffeine the calcium level decreases (left, p = 0.02, Wilcoxon signed rank test with continuity correction). Right: spontaneous activity (measured as standard deviation) increases in the presence of 5 mM caffeine (p = 0.04, Wilcoxon signed rank test with continuity correction, 77 glomeruli from 6 bees). (PDF 207 kb) [file 359_2013_805_MOESM3_ESM.pdf]

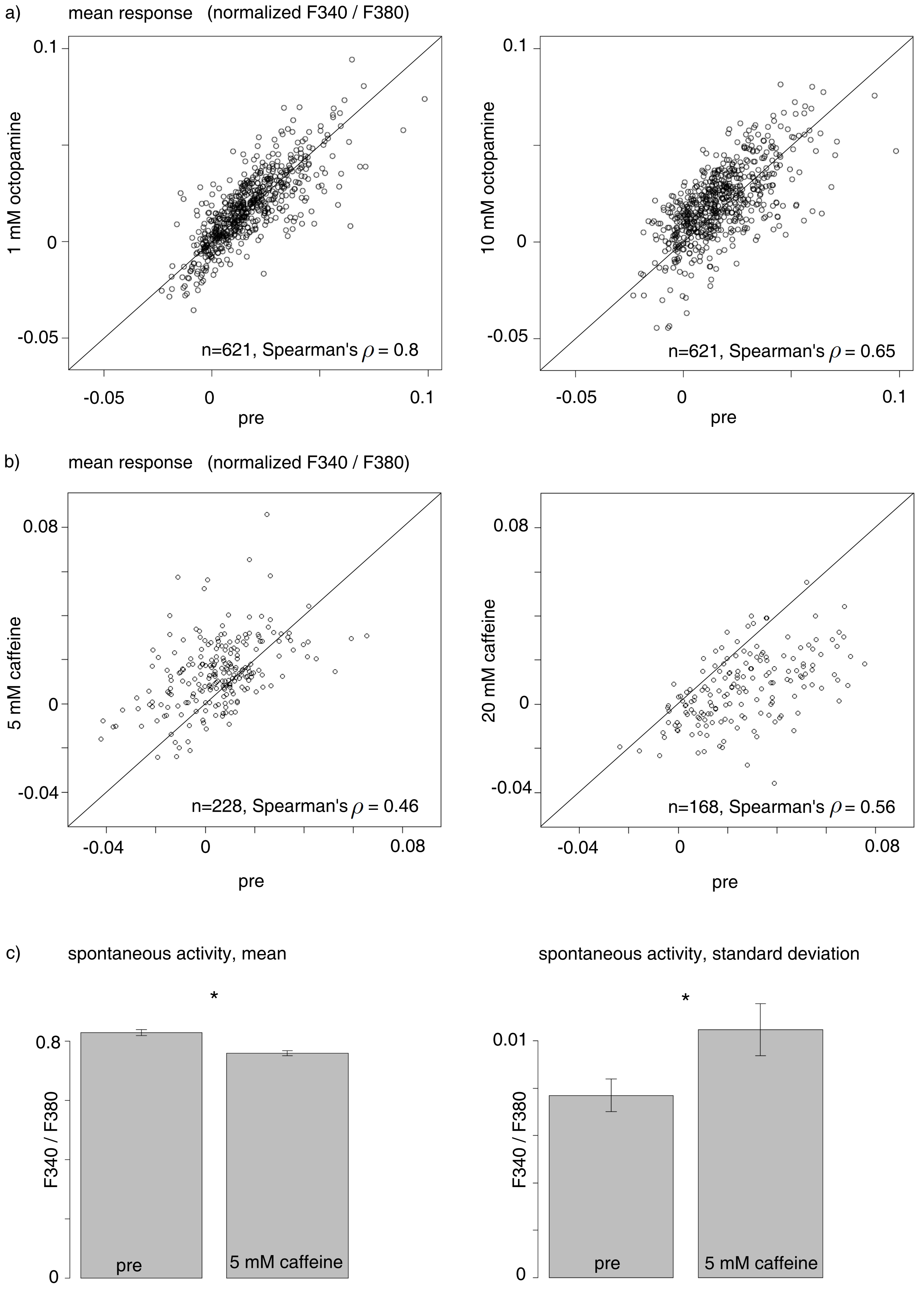
a)
mean response (normalized F340 / F380)
1 mM octopamine
n=621, Spearman's ρ = 0.8
10 mM octopamine
n=621, Spearman's ρ = 0.65
pre
b)
mean response (normalized F340 / F380)
5 mM caffeine
n=228, Spearman's ρ = 0.46
20 mM caffeine
n=168, Spearman's ρ = 0.56
pre
c)
spontaneous activity, mean
spontaneous activity, standard deviation
F340 / F380
pre
5 mM caffeine
*
