# Supplementary material for: Octopamine modulates activity of neural networks in the honey bee antennal lobe
Source: J Comp Physiol A Neuroethol Sens Neural Behav Physiol. 2013 May 17;199(11):947–62. doi: 10.1007/s00359-013-0805-y (PMC3825135; doi:10.1007/s00359-013-0805-y)
Supplement: Supplementary file 4 — Supplemental Figure S4: dsAmoa1 treatment reduces OA receptor density a) Example lanes from a western blot using an antibody against AmOA1 (top row) or tubulin (bottom row) for antennal lobes injected with control dsRNA (left), Amoa1 dsRNA (middle), or bees undergoing surgery alone (right). b) Quantification of the level of AmOA1 protein relative to the amount of tubulin confirms a significant reduction in the level of AmOA1 in the ALs of bees treated with Amoa1 dsRNA 24 h after injection (ANOVA, p = 0.046, nsurgery = 7, ncontrol dsRNA = 6, nAmoa1 dsRNA = 6) (PDF 122 kb) [file 359_2013_805_MOESM4_ESM.pdf]

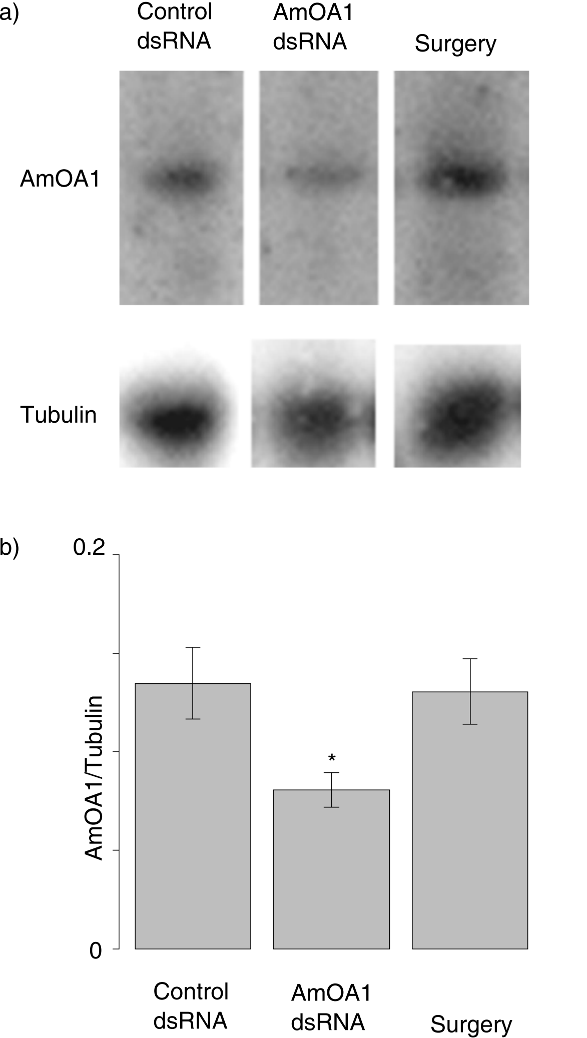
a)
Control dsRNA
AmOA1 dsRNA
Surgery
AmOA1
Tubulin
b)
0.2
0
AmOA1/Tubulin
*
Control dsRNA
AmOA1 dsRNA
Surgery
